# Draft genome sequence of *Psychrobacter* sp. strain TPE 89C isolated from Antarctic seawater

**DOI:** 10.1128/mra.00241-26

**Published:** 2026-06-15

**Authors:** Constanza Griffiths-Sanhueza, Matilda G. Trujillo, Felipe Moraga, Ítalo M. Urrutia, Sebastián Ramirez-Araya, Roberto Bastías, Jorge Olivares-Pacheco, Katherine García, Nicolás Plaza

**Affiliations:** 1 Instituto de Ciencias Biomédicas, Facultad de Ciencias de la Salud. Universidad Autónoma de Chile, Santiago, Chile; 2 Molecular Biology Laboratory, Masblue Research and Innovation Center, Iquique, Chile; 3 Instituto de Biología, Pontificia Universidad Católica de Valparaíso, Valparaíso, Chile; 4 Grupo de Resistencia Antimicrobiana en Bacterias Patógenas y Ambientales, GRABPA, Instituto de Biología, Facultad de Ciencias, Pontificia Universidad Católica de Valparaíso 28047, Valparaíso, Chile; Montana State University, Bozeman, Montana, USA

**Keywords:** *Psychrobacter*, Antarctica, draft genome

## Abstract

We report the draft genome sequence of *Psychrobacter sp*. strain TPE 89C, isolated from Antarctic seawater (25 m depth). The 3.41-Mbp assembly (42.4% GC, 484× coverage) contains a putative Type I-F CRISPR-Cas system with 86 spacers. This resource supports future research on microbial defense and adaptation in extreme environments.

## ANNOUNCEMENT

*Psychrobacter* (family Moraxellaceae) includes Gram-negative, catalase- and oxidase-positive bacteria widely distributed in cold marine ecosystems (1–3). These versatile, osmotic-tolerant psychrotolerant microorganisms inhabit diverse niches, including air, polar seawater, permafrost, and animal-associated sources (4–7). Strain TPE 89C was isolated from seawater (25 m depth) in Greenwich Bay, South Shetland Islands, Antarctica (62.47° S, 59.69° W) during the 60th Chilean Antarctic Expedition (ECA60) (January 2024). This isolate was selected for its ability to grow in 5% NaCl at temperatures between 4°C and 25°C and for phenotypic resistance to penicillin, carbapenems, and linezolid. As antibiotic resistance is frequently associated with mobile genetic elements (MGEs) (8), this genome serves as a model to explore the dynamics between horizontal gene transfer and CRISPR-Cas microbial defense systems.

Seawater was collected using Niskin bottles and filtered through 3-, 0.45-, and 0.22-μm membranes. Strain isolation from the 0.22-μm filter used Marine Broth 2216 (BD Difco, Sparks, MD, USA) solidified with 1.5% agar (BD Difco), followed by growth in Luria-Bertani broth (BD Difco) at 25°C for 24 h. DNA was extracted using the Quick-DNA Fungal/Bacterial Miniprep Kit (Zymo Research, Irvine, CA, USA), quantified with a Fluo-200 fluorometer (AllSheng, Hangzhou, China), and integrity was verified using a Qsep 400 system (BiOptic Inc., New Taipei, Taiwan), with a DNA Quality Number (DQN) of 9.7. Without prior identification, sequencing on the MGI DNBSEQ-G400 platform (MGI Tech, Shenzhen, China) was performed using the MGIEasy Fast FS Library Prep Set V2.0 kit (MGI Tech) (150-bp paired-end). FastQC v0.12.1 (9) confirmed quality (*Q* > 30) for 5,503,335 raw reads. Assembly via Shovill v1.1.0 with SPAdes v4.2.0 (10, 11) used a 500-bp minimum length and Trimmomatic adapter removal (12).

The final assembly spans 3,412,258 bp across 109 contigs, with an *N*_50_ value of 57,566 bp and a 42.4% GC content, as evaluated by QUAST v5.2.0 (13). Final genomic coverage was 484× based on total reads. Completeness and contamination were assessed at 100% and 0.09%, respectively, using CheckM2 v1.1.0 (14). Taxonomic identity was confirmed post-sequencing through phylogenomic analysis using the Type (Strain) Genome Server (TYGS) (15). Digital DNA-DNA hybridization (dDDH) analysis yielded a value of 56.5% (*d*_4_ formula) with *Psychrobacter fozii* CECT 5889 (<70% species threshold). Consequently, the isolate was designated as *Psychrobacter sp*. strain TPE 89C. Functional annotation with PGAP v6.10 (16) identified 2,816 potential coding sequences (CDS), 41 tRNAs, 6 rRNAs, and 1 CRISPR array.

A putative adaptive immune system was detected, including a potential Type I-F CRISPR-Cas system and 86 spacers identified using CRISPRCasFinder web server (https://crisprcas.i2bc.paris-saclay.fr; accessed March 2026) (17). A BLASTn (v2.17.0) (18) search of these spacers against the NCBI viral database (taxid:10239) revealed three significant matches (*E*-value ≤0.03), including two sequences related to *Inoviridae*-like (MH617190.1) and one *Siphoviridae*-like phage (MG252615.1), while 96% of the spacers showed no similarity to currently available viral genomes. In addition, identity matches (100% identity, 17/17 nt) with spacers from *Megamonas funiformis* and *Fibrobacter succinogenes* were observed, suggesting exposure to shared environmental mobile genetic elements rather than direct phylogenetic relationships.

## Data Availability

The draft genome sequence and raw reads of *Psychrobacter* sp. strain TPE 89C have been deposited in the NCBI database under BioProject accession number PRJNA1424545 and BioSample accession number SAMN55386688. The whole-genome shotgun project has been deposited in GenBank under accession number JBVFXW000000000; the version described in this paper is the first. The raw sequencing reads are available in the SRA under accession number SRR37273409.
